# Evaluation of the Acceptability of a Prenatal Program for Women With Histories of Childhood Trauma: The Program STEP

**DOI:** 10.3389/fpsyt.2021.772706

**Published:** 2021-11-04

**Authors:** Nicolas Berthelot, Christine Drouin-Maziade, Julia Garon-Bissonnette, Roxanne Lemieux, Thibaut Sériès, Carl Lacharité

**Affiliations:** ^1^Department of Nursing Sciences, Université du Québec à Trois-Rivières, Trois-Rivières, QC, Canada; ^2^Centre d'études interdisciplinaires sur le développement de l'enfant et la famille, Trois-Rivières, QC, Canada; ^3^CERVO Brain Research Center, Quebec City, QC, Canada; ^4^Interdisciplinary Research Center on Intimate Relationship Problems and Sexual Abuse, Montréal, QC, Canada; ^5^Groupe de recherche et d'intervention auprès de l'enfant vulnérable et négligé, Trois-Rivières, QC, Canada; ^6^Department of Psychology, Université du Québec à Trois-Rivières, Trois-Rivières, QC, Canada

**Keywords:** pregnancy, prevention, child abuse, mentalization, intergenerational, treatment, mothers

## Abstract

**Background:** Childhood trauma would negatively affect pregnant women's mental health and would have intergenerational repercussions. However, there is a paucity of prenatal interventions specifically designed for women exposed to childhood trauma. The study aims to evaluate the acceptability of STEP, a manualized group intervention designed for pregnant women having experienced early life adversity.

**Methods:** The acceptability of STEP was assessed in four phases. In Phase 1, six experts evaluated whether the program activities were pertinent and trauma sensitive. In Phase 2, three parents read the intervention manuals and evaluated whether they considered each session relevant, interesting, and clear. In Phase 3, the program was briefly presented by phone to 309 pregnant women from the community. Women were inquired about their interest in the program, and the reasons for their lack of interest were assessed. In Phase 4, 30 pregnant women exposed to childhood trauma participated in the program and completed anonymous satisfaction questionnaires after each session. Psychological distress was also measured before and after the program.

**Results:** All activities were rated by independent experts as highly pertinent, adequate, and sufficiently safe to be offered to pregnant women. Parents who read through the intervention manuals also considered that the sessions were relevant, clear, and interesting. About half of the pregnant women from the community showed interest in the program. Participants reported very high levels of satisfaction and a significant decrease in psychological distress during the program.

**Conclusions:** Our findings show a high level of convergence among various indicators of program acceptability.

## Introduction

Childhood trauma (here defined as abuse or neglect before 18 years old) is frequent in pregnant women from the community (1, 2). Having a history of childhood trauma may complexify the experience of pregnancy and motherhood since the severity of exposure to trauma has been associated with poorer physical health (3), antenatal attachment (4) and mental health problems (5) during pregnancy, which in turn are predictive of poor postnatal health and adaptation (6). In addition, the phenomenon of childhood trauma would be transgenerational in two distinctive ways. First, children born to a mother exposed to childhood trauma would be three times more likely than offspring of non-exposed mothers to be maltreated themselves (7). Recent, meta-analytic evidence confirmed that a parental history of childhood trauma figures among the most important risk factor for maltreatment (8). Second, even in the absence of a repetition of maltreatment, offspring of parents with a history of trauma are at increased risk of neurobiological (9) and developmental problems (10), as well as of displaying insecure-disorganized attachment patterns (11). This intergenerational transmission of trauma would partly take place during the fetal phase (12). Accordingly, prenatal interventions may have a greater preventive effect than postnatal interventions or may potentialize their effects.

However, intervention research remains scarce in the field of childhood maltreatment (13, 14) and, in spite of 40 years of research evidence on the intergenerational repercussions of childhood trauma, currently there would be little empirically-supported prenatal intervention specifically designed for pregnant women exposed to childhood maltreatment (12, 15). *Survivor's Mom Companion* (SMC) is a self-study psychoeducational program aiming to provide information about how trauma can affect childbearing and mothering, and to teach skills to improve emotion regulation, the management of PTSD symptoms and to reduce interpersonal reactivity (16). Each module is complemented by a brief session with a tutor, a role more akin to that of a teacher than to a therapist (16). The *Perinatal Child-Parent Psychotherapy* [P-CPP; (17)] is a perinatal adaptation of the dyadic psychotherapy developed by Lieberman et al. (18) for mothers exposed to domestic violence or other types of trauma. The P-CPP aims to strengthen pregnant women's emotional attunement, reflective processing and sense of bonding with the baby. It is a psychotherapy that uses complex treatment modalities, such as trauma-informed interpretations, allowing the processing of trauma-related memories (17). Both SMC and the P-CPP appear as promising interventions for pregnant women exposed to early life adversity (19, 20). However, there may be benefits to offering interventions that go a step further in terms of intervention complexity and that are more personalized than psychoeducational programs, and yet that remain sufficiently concise and oriented to facilitate dissemination. Additionally, offering such interventions in groups may have a normalizing effect that facilitates the exploration of attachment representations. Thus, we developed the program STEP (Supporting the Transition to and Engagement in Parenthood), a prenatal group program for women having experienced childhood trauma, and evaluated whether this program was suitable, adequate, safe, and effective according to the perspective of experts and of the targeted population.

**Figure 1:**
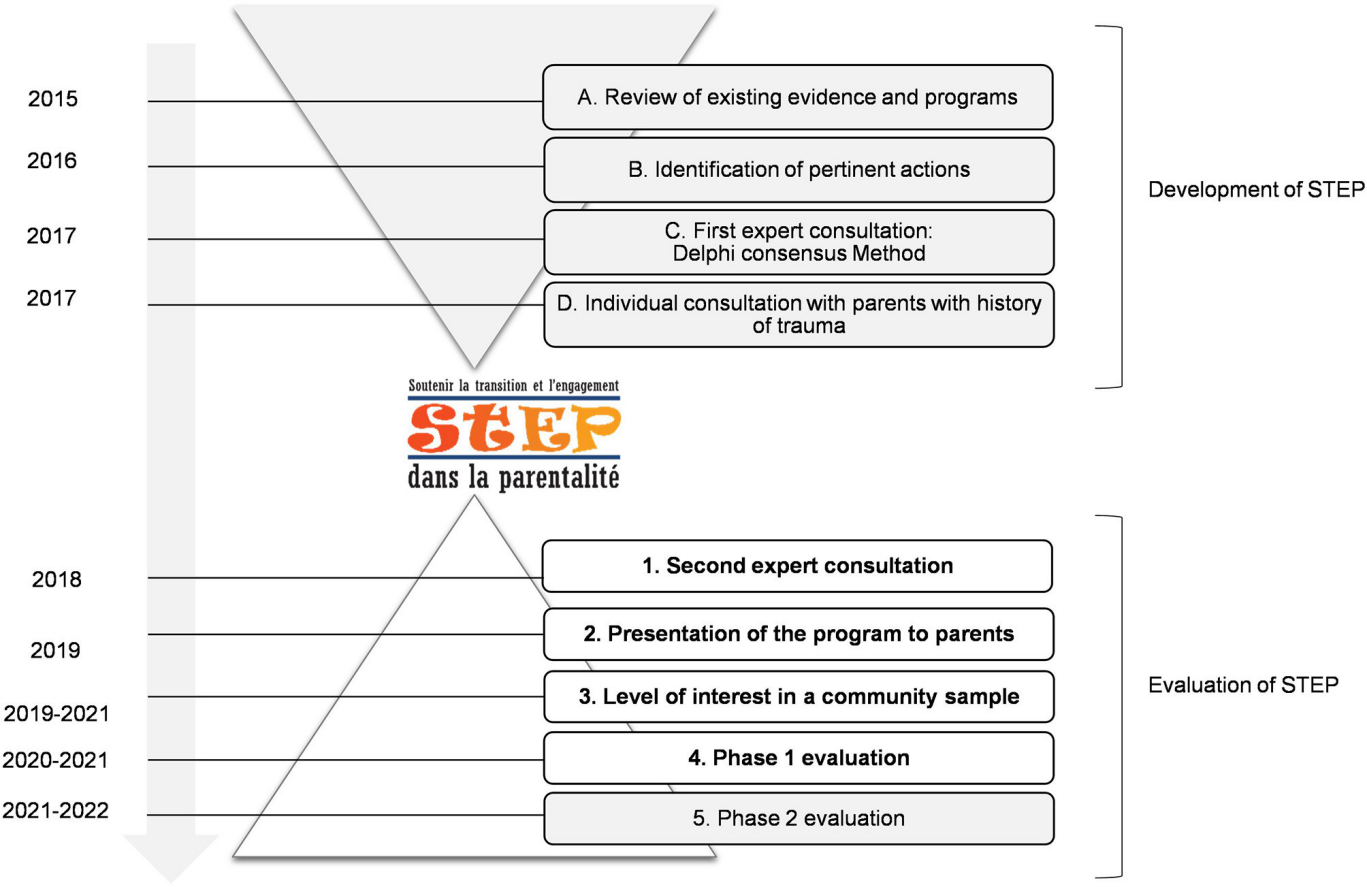
Timeline of the development and evaluation of STEP. In the current study, results of phases 1–4 are presented.

To our knowledge, STEP is the first prenatal group program specifically designed for pregnant women having experienced childhood maltreatment or other types of complex trauma. The program was developed and evaluated in nine successive phases, as described in Figure 1. After reviewing existing evidence regarding the impacts of childhood trauma during the perinatal period (phase A), the developers of the program (NB, CD-M, RL) identified a preliminary list of 36 actions that may be relevant to achieve for pregnant mothers having experienced childhood trauma (phase B). In phase C, 15 stakeholders representing nine different health care and community organizations working with families or trauma survivors were invited to participate in a Delphi consensus process during which they coded how important they considered each pre-identified action and whether they were already conducting similar interventions in their clinical setting. Two central clusters of actions were identified: actions aiming to support mentalization about self and parenthood, and actions aiming to support mentalization of trauma (21). This consultation process was complemented by semi-structured interviews with 10 parents, each with a preschool child, who identified themselves as having a history of childhood maltreatment (phase D). The parents further clarified their needs and stated that they would have greatly appreciated to having had the opportunity to participate in a trauma-informed group program during pregnancy (22). Based on the observations of these four preliminary phases, the program was developed and manualized.

The general goals of STEP are to foster emotion regulation and reflective capacities in pregnant women exposed to early life adversity, in order to (a) sustain maternal well-being during pregnancy and the years following delivery, (b) support maternal bonding and healthy development in offspring, and (c) contribute to interrupting intergenerational cycles of childhood maltreatment. STEP reaches these goals by equipping participants with knowledge regarding childhood trauma, a better understanding of the impact of trauma on their current functioning, abilities to regulate emotions in stressful situations, interactions with other women having experienced adversity, and connections with existing resources in the community. STEP consists of eight or nine sessions of 2-h, is manualized and is designed to be offered by two facilitators to groups of four to six pregnant women. The program was originally conceived to be offered face-to-face, but has been offered online during the COVID-19 pandemic. The intervention is considered a “trauma-informed accompaniment program” during which facilitators share information, animate reflective activities, facilitate exchanges, and focus on the participants' experience in the “here and now.” The program is mainly based on the paradigm of trauma and violence-informed practices (23) and on mentalization theory (24).

STEP is divided into three sections. The first section, entitled “*Becoming a Mother*,” aims to explore and normalize the emotions experienced by the participants in the course of their pregnancy and to support the use of healthy emotion regulation strategies. The second section, entitled “*A Look at My Own History*,” aims to support mentalization of trauma by discussing the nature of trauma and its impact; by validating the participants' feelings as understandable responses to trauma; by supporting a reflection on positive and harsh experiences with significant others and the ways both types of experiences have influenced current mental states and behaviors; and by identifying how participants coped with trauma. The last section, entitled “*Looking Ahead*,” aims to explore participants' needs and strengths, to present available resources to support resilience and to provide the opportunity to envision positive and challenging moments with the child.

The current study aims to evaluate the acceptability of STEP by assessing whether its specific contents and activities are coherent with the experience of pregnant women exposed to childhood trauma. We did so by (1) evaluating whether the activities of the program are trauma-informed and appropriate according to experts and parents; (2) evaluating the level of interest in the program in a community sample of pregnant women; (3) providing preliminary data on the short-term changes in psychological distress in women participating in STEP; and (4) exploring the mothers' participation experience in the program.

## Materials and Methods

### Participants and Procedure

The acceptability of STEP was assessed in four successive phases (see Figure 1). In Phase 1, the intervention was presented to a panel of six independent experts. Among the experts were clinician-researchers in psychology, perinatal nurses and midwives having complementary expertise in child maltreatment, neglect, perinatal care, group therapy, program evaluation, motherhood, women mental health, fatherhood, couple relationship, personality disorders, and domestic violence. In Phase 2, three parents who had experienced adverse life events (two mothers and one father) read the intervention manuals and evaluated each session. In Phase 3, the program was briefly presented by phone to 309 pregnant women from the community recruited in prenatal clinics. They were asked whether they would like to participate in STEP if they were found eligible. When the facilitators were available to offer a new group, they contacted the participants who reported being interested in the program, completed no more than 28 weeks of gestation and reported significant trauma (see Measures section) to invite them to participate in Phase 4. This permitted to recruited 30 pregnant women exposed to childhood trauma (see Table 1) who participated in and evaluated the program (Phase 4).

**Table 1:** Demographics of pregnant women who participated in the STEP program (*N* = 30).

**Demographic characteristics**	**Participants (*N* = 30)**
Age, mean (SD), range	28.73 (5.0), 21–43
Primiparous, *n* (%)	22 (75.9)
**Relationship status**, ***n*** **(%)**
Married or in common-law relationship	17 (56.7)
In relationship	11 (36.7)
Single mother	2 (6.7)
**Education status**
High school diploma or less	3 (10.0)
Post-secondary education (collegial or professional training)	17 (56.7)
University degree	10 (33.3)
**Annual income**	
Annual income level below the low-income status^a^, *n* (%)	8 (26.7)
**Childhood trauma exposure**
CITC score, mean (SD)^b^	8.73 (4.8)
Trauma classification on the CTQ, *n* (%)^c^	19 (63.3)
Physical abuse, *n* (%)	2 (6.7)
Sexual abuse, *n* (%)	10 (33.3)
Emotional abuse, *n* (%)	16 (53.3)
Physical neglect, *n* (%)	7 (26.3)
Emotional neglect, *n* (%)	5 (16.7)

a *The low-income cutoff for a family with one child is Can$ 33,396*.

b *The CITC assesses 34 potentially traumatic events during childhood or adolescence. All participants were classified as having been exposed to trauma according to the CITC (≥1 experience of abuse or neglect or ≥3 potentially traumatic experiences)*.

c *The CTQ assesses five types of childhood trauma with validated cut-offs for each subscale: physical abuse (≥8), sexual abuse (≥8), emotional abuse (≥10), physical neglect (≥9), and emotional neglect (≥15) (25). Participants were considered as having experienced trauma when they scored higher than the cut-off on at least one scale*.

### Measures

#### Program Acceptability

Evaluation of the acceptability of STEP was measured throughout four successive phases. In Phase 1, all program activities were presented to the panel of independent experts during a full day of a face-to-face meeting. After the presentation of each activity, experts were required to evaluate whether it was pertinent and trauma-sensitive using a 10-point Likert scale ranging from 1 (totally disagree) to 10 (totally agree). In Phase 2, three parents from our target population read the intervention manuals and evaluated whether they considered each session relevant, interesting, and clear, using a 3-point scale (weak, moderate, strong). They also used a voice recorder to record all their comments as they occurred while reading the manuals. This was successively used by the team to refine the program, but this qualitative information is not reported in the current manuscript. In Phase 3, after a brief presentation of the program, the pregnant women who did not wish to participate in STEP were asked to choose one of the following 5 reasons for their refusal: (a) not feeling the need, (b) being unavailable, (c) being held back by the group format, (d) not wanting to think about past traumas, (e) or other reasons. Finally, in Phase 4, the pregnant women who participated in the program anonymously reported on their appreciation of each session, right after the sessions. They were invited to indicate to what extent they considered the session (a) useful and (b) emotionally challenging, and whether they (c) learned things, (d) had significant insights, and (e) were motivated to participate in the next sessions, using 5-point Likert scale ranging from 1 (Totally disagree) to 5 (Totally agree). At the end of the program, five questions were added to evaluate whether they observed positive changes in the way they felt about themselves, their past and motherhood; considered having learned helpful things throughout the program; and identified particular strengths they had as mothers.

#### History of Childhood Maltreatment

Participants were considered eligible for the program if they had experienced trauma, according to the Childhood Trauma Questionnaire [CTQ-28; (26)] and the Childhood Interpersonal Traumas Checklist [CITC; (27)]. The CTQ includes 28 items and evaluates five types of maltreatment using a five-point Likert scale ranging from 0 (never true) to 5 (always true). The CTQ shows good validity across clinical and general populations (26). The CITC is a self-reported measure assessing the occurrence, during childhood or adolescence, of 34 experiences that are considered definitely abusive or neglectful (ex. sexual abuse, not having enough food, being physically disciplined) or as being potentially traumatic (parental mental illness, role reversal, absence of proper boundaries in the family). The CITC has good psychometric properties in samples of pregnant women (27). The cut-offs of the instruments are reported in Table 1. We previously showed in a larger sample of pregnant women that the CTQ has a good consistency (10).

#### Psychological Distress

Changes in psychological distress was measured with the French version of the Kessler Psychological Distress Scale (K10) (28, 29) administered before the intervention (around the beginning of the 2nd trimester) and at 36-weeks of pregnancy. The K10 has 10 items and inquires about anxiety (i.e., “how often did you feel so nervous that nothing could calm you down?”) and depressive symptoms (i.e., “how often did you feel so sad that nothing could cheer you up?”) experienced by the participant during the last month using a 5-point Likert scale ranging from 1 (None of the time) to 5 (All of the time). Higher scores indicate more symptoms and distress. Both the English and French versions have similarly satisfactory psychometric properties (28) and the instrument has been shown to screen adequately for mood and anxiety disorders in pregnant women (30, 31). We previously showed in a larger sample of pregnant women that the K10 has a good consistency (31).

## Results

**Figure 2:**
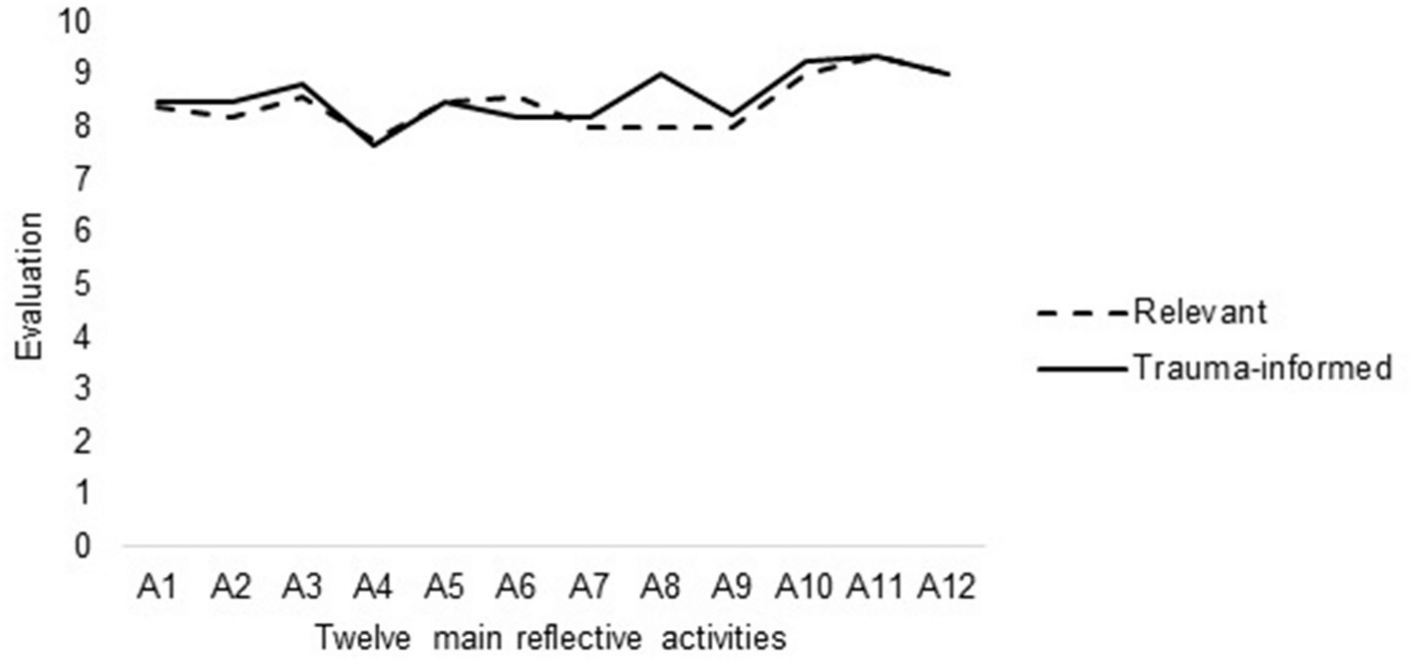
Evaluation of the twelve main reflective activities of the program by a panel of independent experts. Six independent experts rated the twelve main activities of the program using a 10-point Likert scale ranging from 1 (Totally disagree) to 10 (Totally agree).

Results of the consultation process with six independent experts (Phase 1) confirmed that all activities were pertinent, adequate and trauma-informed (Figure 2). Indeed, the 12 main reflective activities proposed during the sessions were all rated as very likely to reach their goal (average M = 8.45, SD = 0.49) and as being at low-risk of creating distress and re-traumatizing participants (average M = 8.60, SD = 0.50).

In Phase 2, all parents confirmed the relevance of the program and its activities. The core seven sessions of STEP (excluding the introductory and final sessions) were all rated as relevant (M = 3.00, SD = 0.00), clear (M = 2.91, SD = 0.16) and interesting (M = 2.83, SD = 0.30).

In Phase 3, 52.75% of pregnant women were interested in participating in the program. Reasons for refusal were: not feeling the need (49.32%), being unavailable (21.92%), being held back by the group format (12.33%) or not wanting to think about past traumas (0.68%). The remaining participants (15.75%) did not provide a motive. Of the 163 interested participants, 89 (54.60%) reported significant childhood adversity. In sum, our data suggest that around 28.8% of pregnant women from the community would be interested, engaged and eligible for the program.

**Figure 3:**
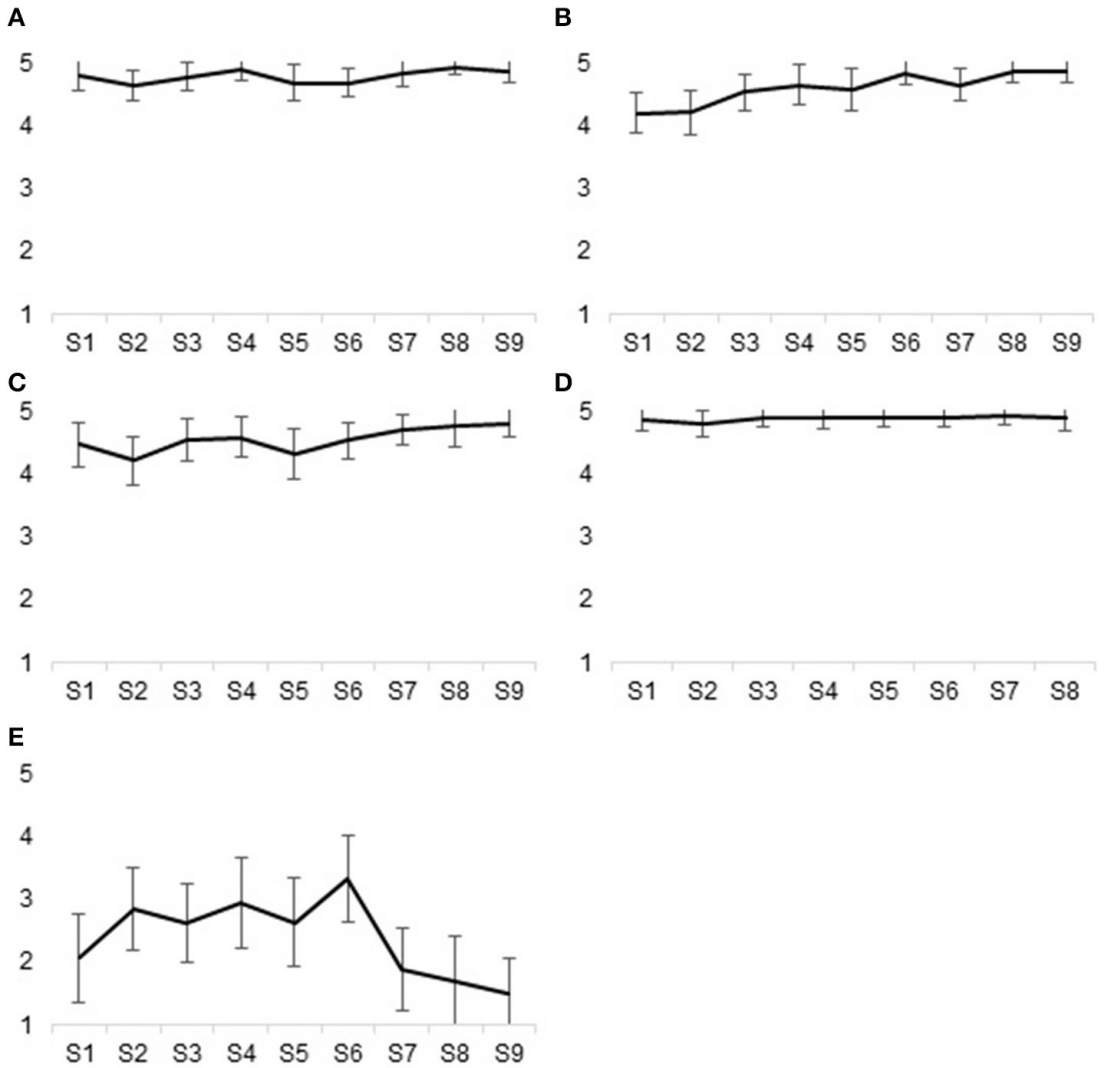
Level of satisfaction reported after each session of the program according to five indicators. Thirty pregnant women who participated in the program evaluated to what extent they considered each of the nine sessions to be useful, to be informative, to stimulate new insights, to be motivating, and to be emotionally challenging using a five-point Likert scale ranging from 1 (Totally disagree) to 5 (Totally agree). Length of the error bars represent the 95% confidence interval for the mean. **(A)** Useful, **(B)** Informative, **(C)** New insights, **(D)** Motivating, **(E)** Emotionally challenging.

**Figure 4:**
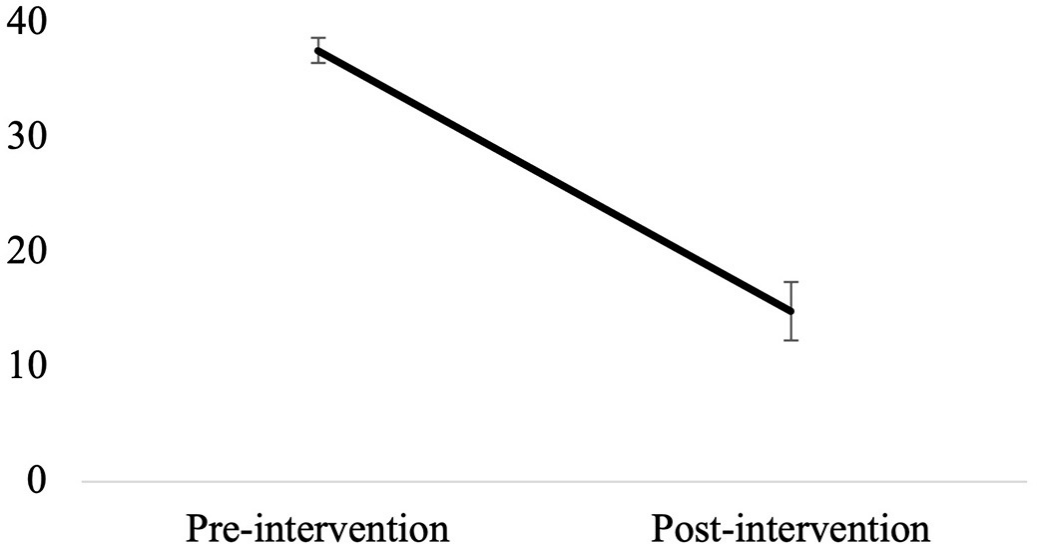
Evolution of psychological distress between the beginning of the program and post-intervention assessment at the end of pregnancy. Error bars represent the standard error for the mean.

In Phase 4, seven groups of pregnant women exposed to childhood trauma participated in the program, for a total of 30 participants. One participant had to quit because of medical complications requiring medical appointments taking place at the same time as the sessions. Another participant dropped out after the first session. All other participants completed the program and attended, on average, 94% of the sessions. As shown in Figure 3, all sessions were rated as useful (M = 4.80, SD = 0.43), informative (M = 4.63, SD = 0.57), developing new insights (M = 4.35, SD = 0.61), and motivating (M = 4.88, SD = 0.32). The level of emotional arousal during the sessions of the program was generally optimal (M = 2.16, SD = 1.35), i.e., not too high nor too low. The sessions addressing past traumas (sessions 5 and 6) were not considered overly emotionally challenging (M = 2.69, SD = 1.40). Overall, satisfaction regarding the program was very high: all participants reported positive changes in the way they felt about themselves (M = 4.91, SD = 0.29), their past (M = 4.87, SD = 0.34), and motherhood (M = 4.96, SD = 0.22) since the beginning of the program. Also, they all acknowledged having learned helpful things (M = 4.96, SD = 0.21) and being more aware of their strengths as mothers (M = 4.83, SD = 0.39). The paired sample *t*-test showed significant improvements in terms of psychological distress between the beginning of the program (M = 38.57, SD = 5.48) and the post-intervention assessment at the end of pregnancy (M = 14.84, SD = 12.88), *t*_(25)_ = 7.78, *p* = < 0.001 (Figure 4).

## Discussion

The present study offers primary data on the acceptability of STEP, one of the first prenatal group interventions for women exposed to childhood trauma. STEP aims to support mentalization about self, parenthood and past traumas in pregnant women in order to support maternal well-being and mitigate the intergenerational repercussions of trauma. Our results showed that STEP has a high level of acceptability, both from the point of view of experts and of the targeted population.

Addressing trauma during pregnancy could have important benefits, but could also increase distress, and little is known about the safety of addressing trauma during pregnancy since pregnant women are frequently excluded from clinical trials addressing post-traumatic stress disorder (32). Therefore, we wanted to evaluate the acceptability of the program according to different indicators, including its relevance and its safety. Overall, all activities were rated by independent experts as highly pertinent, adequate, and sufficiently safe to be offered to pregnant women. Parents who experienced adversity and who read through the intervention manuals had similar perceptions and considered all sessions as relevant, clear, and interesting. In addition, we had the opportunity to evaluate whether the program appeared appealing and was likely to reach a significant number of women because of a longitudinal study we were conducting with a sample of pregnant women from the community. Results showed that about half of the pregnant women were interested in the program. Thus, we offered the program to seven groups of pregnant women. Overall, only one participant purposely dropped out, and very high levels of satisfaction were reported by participants after each session of the program. A significant decrease in psychological distress was also observed between the beginning and the end of the program. Since prenatal distress was shown to be associated with poorer postnatal mental heath and offspring development (33, 34), these results are promising. However, it remains to be determined whether the decrease in psychological distress we observed during the program is more important than what would have been observed in pregnant women receiving usual prenatal care.

Because it relies primarily on a quantitative perspective of the acceptability of a program, this study does not describe a set of factors that contribute to a deeper understanding of the acceptability of STEP. A new study based on a qualitative design would enable us to explore this issue and to describe various profiles of response to the program. Clinical trials, including a control group of women receiving usual prenatal care and postnatal measurements, will also need to be conducted to evaluate the efficiency of the program in supporting maternal well-being, reflective capacities, maternal engagement, and infant development, as well as mechanisms of change.

Overall, our findings show a high level of convergence among various indicators of program acceptability. Based on these results, it is possible to conclude that STEP is an efficient program in terms of the quality of response to the specific needs of pregnant women who have experienced trauma in their childhood. Such trauma-informed prenatal interventions may have important implications for maternal health, child development and public health, considering the well-known repercussions of maternal trauma on the experience of childbearing, maternal mental health, postnatal adjustment, and child development (35).

## Data Availability Statement

The raw data supporting the conclusions of this article will be made available by the authors, without undue reservation.

## Ethics Statement

The studies involving human participants were reviewed and approved by the Comité d'éthique de la recherche du Center intégré universitaire de santé et de services sociaux de la Mauricie-et-du-Centre-du-Québec (CER-2016-016) and the Comité d'éthique de la recherche avec des êtres humains de l'Université du Québec à Trois-Rivières (CER-16-226-10). The patients/participants provided their written informed consent to participate in this study.

## Author Contributions

NB, CD-M, and RL conceptualized and designed the study, supervised material preparation and supervised data collection. JG-B performed and coordinated data collection and analyses. NB wrote the first draft of the manuscript. CL, CD-M, and TS revised and reviewed the manuscript for important intellectual content. All authors contributed to data interpretation, commented on previous versions of the manuscript, read, and approved the final manuscript.

## Funding

The research leading to these results received funding from the Public Health Agency of Canada (Grant No. 1617-HQ-000015) and the Canada Research Chairs (Grant No. 950-232739).

## Conflict of Interest

The authors declare that the research was conducted in the absence of any commercial or financial relationships that could be construed as a potential conflict of interest.

## Publisher's Note

All claims expressed in this article are solely those of the authors and do not necessarily represent those of their affiliated organizations, or those of the publisher, the editors and the reviewers. Any product that may be evaluated in this article, or claim that may be made by its manufacturer, is not guaranteed or endorsed by the publisher.
